# Lead SARS-CoV-2 Candidate Vaccines: Expectations from Phase III Trials and Recommendations Post-Vaccine Approval

**DOI:** 10.3390/v13010054

**Published:** 2020-12-31

**Authors:** Ebenezer Tumban

**Affiliations:** Texas Tech University School of Veterinary Medicine, Amarillo, TX 79106, USA; etumban@ttu.edu; Tel.: +1-806-834-0472

**Keywords:** SARS-CoV-2, COVID-19, SARS-CoV-2 vaccines, clinical trials

## Abstract

Severe acute respiratory syndrome coronavirus 2 (SARS-CoV-2) is transmitted primarily through respiratory droplets/aerosols and it causes COVID-19. The virus infects epithelial cells by using the spike protein on its surface to bind to angiotensin-converting enzyme 2 receptor on the cells. Thus, candidate vaccines targeting the spike protein are currently being developed to prevent against infections. Approximately 44 SARS-CoV-2 candidate vaccines are in clinical trials (phase I–III) and an additional 164 candidates are in preclinical stages. The efficacy data from phase I/II trials of lead candidate vaccines look very promising with virus-neutralizing geometric mean antibody titers in the range of 16.6–3906. Most recently, two SARS-CoV-2 candidate vaccines, BNT162b2 and mRNA-1273, have been granted the first emergency use authorization (EUA) in the U.S.; BNT162b2 has also been granted an EUA in the United Kingdom, Canada, and in the European Union. This review assesses whether SARS-CoV-2 candidate vaccines (with approved EUA or in phase III trials) meet the criteria for an ideal SARS-CoV-2 vaccine. The review concludes with expectations from phase III trials and recommendations for phase IV studies (post-vaccine approval).

## 1. Introduction

SARS-CoV-2 was first reported, in late 2019, in humans in Wuhan (China); the virus is believed to have been transmitted to humans from an unknown animal reservoir [1,2,3]. Since then, the virus has spread worldwide; as of 1st December 2020, >63.5 million people have been infected with the virus, with >1.4 million deaths [4]. SARS-CoV-2 is suspected to have evolved from a bat coronavirus, RaTG13 [5]; the spike protein of SARS-CoV-2 is 97.4% identical to that of RaTG13, while it is only 76% and ~35% identical to that of SARS-CoV and the middle east respiratory syndrome (MERS) coronavirus, respectively. SARS-CoV-2 is transmitted primarily through respiratory droplets/aerosols. The virus causes COVID-19, which is associated with flu-like symptoms (fever, chills, cough; 83% of patients), pneumonia (31% of patients), acute respiratory destress syndrome (17% of patients), nausea/vomiting (1% of patients), and diarrhea (~2% of patients) [6,7]. More recently, the virus has been linked to stroke and pediatric multi-system inflammatory syndrome in children [8,9,10,11]. COVID-19 disproportionately affects racial/ethnic minorities; this group has the highest number of people with underlying health conditions (comorbidities) such as obesity, diabetes, heart disease, and HIV [12]. Thus, a racial/ethnic minority person is 2.1 times more likely to die from COVID-19 than a non-ethnic minority person [13].

SARS-CoV-2 infects epithelial cells by using the spike protein on its surface to bind to angiotensin-converting enzyme 2 receptor on the cells [14,15,16]. Following binding to the receptor, the spike protein interacts further with cellular proteases (e.g., furin, transmembrane protease serine 2, and cathepsin L) on the cell, which cleave the spike protein, thus allowing the virus to fuse with the host cell membrane to enter the cell [17]. The viral genome (positive-sense single-stranded RNA) including nucleocapsid are then released into the cytoplasm. The viral genome (non-structural proteins) is translated, first, by host cell machinery to give rise to RNA-dependent RNA polymerase (RdRp), helicase, and viral proteases. RdRp is then used to replicate the viral genome followed by translation of structural proteins (nucleocapsid protein, spike protein, envelope protein, and membrane protein).

A number of drugs are currently being explored to treat COVID-19 [18]. Recently, the U.S. Food and Drug Administration (FDA) approved Veklury (remdesivir) to treat COVID-19 patients who are ≥12 years old (and weigh at least 40 kg) [19]. Remdesivir targets RdRp, thus preventing the replication of the viral genome. The FDA has also issued an emergency use authorization (EUA) for an investigational neutralizing monoclonal antibody (bamlanivimab) to treat COVID-19 patients in the same age/weight category [20]. The monoclonal antibody targets the spike protein and prevents binding/entry of the virus into the cell. In addition to these, convalescent sera from patients who have recovered from COVID-19 [21,22,23,24] are currently being used to treat patients with SARS-CoV-2 (in early stages of the disease) worldwide. Convalescent sera treatments have led to normalization in body temperature of infected patients, reduction in viral load (negative within 7–12 days of transfusion), reduction in hospitalization time as well as stabilization of patients suffering from respiratory failure [21,22,23,24].

The scientific community and the biotechnology industry have been working relentlessly to develop prophylactic vaccines to prevent against SARS-CoV-2 infections. Two SARS-CoV-2 candidate vaccines (BNT162b2 and mRNA-1273) have recently been granted an EUA after phase II/III trials; BNT162b2 and mRNA-1273 have been granted an EUA in the U.S. [25,26]. BNT162b2 has also been granted an EUA in the United Kingdom [27], Canada, the European Union [28], the United Arab Emirates, Saudi Arabia, etc. In addition to these, the European Union will hold a meeting on 6th January 2021 to consider an EUA application for mRNA-1273 [29]. Moreover, other SARS-CoV-2 candidate vaccines are under phase III trials. An ideal SARS-CoV-2 vaccine, to fight the pandemic, should have the following features:

(i) Elicit long-lasting protective immune responses;
(ii) Should be given to everyone regardless of comorbidity or age, immune status, pregnancy/breastfeeding status;
(iii) Lack the potential to cause antibody dependent enhancement (ADE) or pulmonary immunopathology;
(iv) Should be thermostable in order to enable transportation and storage in developing countries with poor refrigeration facilities;
(v) Be highly immunogenic in the general population including a population with pre-existing anti-vector antibodies.

This review paper reviews data from phase I/II trials of lead candidate vaccines (Table 1) and assesses whether SARS-CoV-2 vaccine with EUA or in phase III trials including those already licensed, prior to phase III trials, meet the criteria for an ideal SARS-CoV-2 vaccine. This review concludes with expectations from phase III trials and recommendations for phase IV studies (post-vaccine approval).

## 2. SARS-CoV-2 Candidate Vaccines with Approved EUA

### 2.1. The BNT162b2 Vaccine

On 2nd December 2020, the United Kingdom granted the first (after phase III trial) EUA for a SARS-CoV-2 vaccine, BNT162b2. BNT162b2 was developed by Pfizer and BioNTech and it is composed of an mRNA of the full length of the spike protein encapsulated in a lipid nanoparticle. The efficacy data in humans that led to its approval are summarized as follows: first, a phase I trial was conducted with 90 healthy participants (18–55 years old); the participants (12 in each group) were immunized twice with 10, 20, or 30 μg/dose of BNT162b2 while other participants (3 in each group) were immunized with a placebo [33]; the second dose was given three weeks after the first dose and neutralization antibody titers were assessed one week after the second dose. Participants immunized with 10, 20, or 30 μg had virus-neutralizing geometric mean antibody titers (NGMATs) of 157, 363 and 361, respectively. Two weeks after the second dose, another neutralization assay was conducted; NGMATs declined slightly to 97, 292, and 163, respectively. The responses decreased further with age; in older adults (65–85 years old; three groups of participants with 12 in each group) immunized with the same doses/schedule, the viral NGMATs after one week were 79, 84, and 149, respectively. Two weeks after the second immunization, NGMATs were 111, 81, and 206, respectively. The NGMATs were within the range of those in convalescent sera from 18–83-year-old patients who have recovered from COVID-19 (NGMAT 90–618; average 94) that were tested alongside with sera from vaccinees.

Data from phase II/III studies have not yet been published. However, Pfizer, in a press release, recently shared some preliminary data from phase II/III studies, which included >43,000 participants globally [34]; 42% of the participants were from diverse racial/ethnic backgrounds and 41% of them were 56–85 years old. Pfizer highlighted that BNT162b2 was well tolerated across all populations with mild or moderate adverse effects (fatigue and headache) that were short lived. Additionally, they highlighted that the vaccine was >94% effective, one week after a second dose of 30 μg. Eight versus 162 cases of COVID-19 were diagnosed in the vaccine and placebo groups, respectively; additionally, 1 case of severe COVID-19 versus 9, were diagnosed in the vaccine and placebo groups, respectively. They went further to highlight that the vaccine was efficacious across age groups (including those >65 years old), gender, and race/ethnicity.

As mentioned above, an EUA for BNT162b2 has been granted in the United Kingdom, Canada, the U.S., the European Union, the United Arab Emirates, Saudi Arabia, etc. It was also mentioned earlier that the data for phase III trials have not yet been peer reviewed for publication and some unknowns were not addressed in the press release in November 2020; thus, the scientific community hopes the following concerns will be addressed (in order to make BNT162b2 an ideal SARS-CoV-2 vaccine) when the data are published:

(i) We do not know whether NGMATs in children (12–17 year) and adults (18–65 years) are similar to those of older adults (>65 years old). It is also not clear whether the vaccine will be tested in children <12 years old.
(ii) We do not know the NGMAT beyond one week after the second immunization; this is very important because in phase I trial, there was a slight reduction in NGMAT, two weeks after the second immunization [33].
(iii) The proportion of participants in the whole trial that were used to assess the efficacy of >94% is not known.
(iv) It is not clear whether the 162 cases of COVID-19 (and 9 severe cases) detected in the placebo group were within a certain age group, race/ethnicity, or had prior SARS-CoV-2 infection.
(v) It is not clear whether the vaccine was tested in participants with comorbidities, which is a group in dire need of a COVID-19 vaccine.

### 2.2. The mRNA-1273 Vaccine

On 18th December 2020, the U.S. granted a second EUA for a SARS-CoV-2 candidate vaccine, mRNA-1273. mRNA-1273, developed by Moderna, is similar to BNT162b2; it is composed of an mRNA of the full length of the spike protein encapsulated in a lipid nanoparticle. In a phase I trial conducted with 45 healthy participants (18–55 years old and >56 years old), two groups of participants (15 in each group) immunized twice with 25 or 100 μg/dose of mRNA-1273 had NGMATs of 339.7 and 654.3, respectively; the second dose was given four weeks after first dose and neutralization assays were conducted 2 weeks after the second dose. The NGMATs were similar or superior to those in convalescent sera from patients who have recovered from COVID-19 [35]. The phase I trial was then expanded to include 40 older adults (56–70 years and ≥71 years). A mixed trend of NGMATs were observed in older adults. NGMATs in ten 56–70-year-old participants and in ten ≥71-year-old participants, immunized with 100 μg/dose (same schedule as in 18–55 years old), were 878 and 317, respectively [36]. Neutralizing antibody titers from a 250 μg dose (in 18–55 years old) are pending.

Moderna, in a press release, has recently shared some preliminary data from phase II/III studies with 30,000 participants (>18 years) in the U.S. [37]. The study included 37% of participants from diverse racial/ethnic minority backgrounds; it also included 42% of participants who are at high risk for severe COVID-19 (i.e., were greater than 65 years old or less than 65 years old but had comorbidities). In the press release, Moderna highlighted that mRNA-1273 was well tolerated across all populations with mild or moderate adverse effects (fatigue, myalgia, arthralgia, headache, pain, and swelling at injection site) that were short lived. The press release further highlighted that mRNA-1273 is 94.5% effective, two weeks after a second dose of 100 μg. Five versus 90 cases of COVID-19 were diagnosed in the vaccine and placebo groups, respectively; 15.8% of the COVID-19 cases were adults >65 years old and 21.1% were from diverse racial/ethnic minority backgrounds. Further, 11 cases of severe COVID-19 were present in the placebo group but no case was present in the vaccine group. The data have not yet been peer reviewed for publication and some unknowns were not addressed in the press release; thus, the scientific community hopes the following concerns will be addressed (in order to make mRNA-1273 an ideal SARS-CoV-2 vaccine) when the data are published:

(i) We do not know whether the participants in the vaccine group who had COVID-19 or severe cases of the disease had lower NGMATs compared to those that did not have the disease. Thus, data comparing NGMAT in these two groups are warranted. It would also be nice to know the percentage of adults, 18–55 years old, in the vaccine group that had COVID-19 compared to older adults, 56–70 years old. This is very important because in phase I trial, NGMAT in older adults (immunized with same dose of antigen/schedule as in phase II/III) seemed to be slightly higher (878) [36] compared to those in 18–55 years old (654.3) [35].
(ii) We do not know what percentage of participants with comorbidities had COVID-19 (if any) in the vaccine group.
(iii) Data on the percentage of individuals that were seropositive, for SARS-CoV-2, after the second dose are not known. This information is very important because it will shed light on how many participants within a group (population) will respond, immunogenically, to the vaccine.
(iv) We do not know whether the NGMAT will change three weeks (and beyond) after the second immunization and/or whether the number of COVID-19 cases in the vaccine group will increase. So far, we have data only on the number of COVID-19 cases, two weeks after the second dose.
(v) The proportion of participants in the whole trial that were used to assess the efficacy of 94.5% is not known. A larger proportion size will reflect reliable data and vice versa.
(vi) Although the study included participants from diverse racial/ethnic backgrounds, the study was conducted only in one country (the U.S.) and did not include children and young adults < 18 years old. Data from other geographic regions around the world and from an age group < 18 years are needed.

## 3. SARS-CoV-2 Candidate Vaccines in Phase II/III Trials

**Figure 1:**
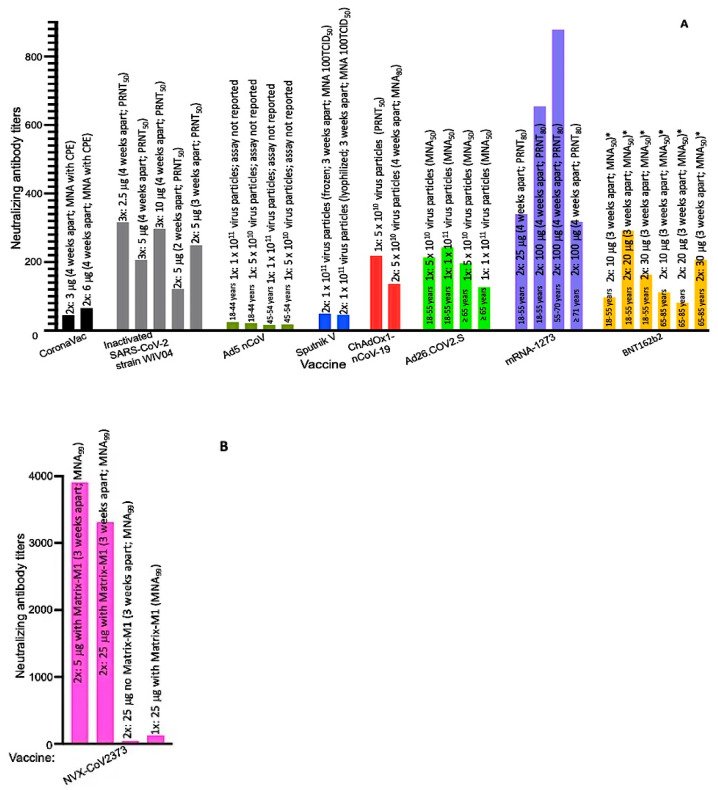
NGMATs of SARS-CoV-2 candidate vaccines in phase III trials. (**A**) NGMATs of CoronaVac, inactivated SARS-CoV-2, Ad5 nCoV, Sputnik V, ChAdOx1 nCoV-19, Ad26.COV2.S, mRNA-1273, and BNT162b2. (**B**) NGMATs of NVX-CoV2373. Neutralization assays were performed using wild-type virus except with the BNT162b2 vaccine (* a reporter SARS-CoV-2 virus expressing mNeonGreen was used). Tissue culture infectious dose (TCID).

As of 18 November 2020, ~44 SARS-CoV-2 candidate vaccines were in clinical trials (phase I–III) and an additional 164 candidates were in preclinical stages [38,39]. These candidate vaccines, developed using different technologies/approaches, include live attenuated SARS-CoV-2, inactivated SARS-CoV-2, replication-deficient vectors expressing the full-length spike protein, replication-competent vectors expressing the full-length spike protein, mRNA of the full-length spike protein, DNA of the full-length spike protein, the full-length recombinant spike protein, the receptor binding domain of the spike protein, SARS-CoV-2 virus-like particles (VLPs), a multi-peptide cocktail of SARS-CoV-2, VLPs displaying receptor binding domain (reviewed in [40,41]). Of the ~44 candidate vaccines in clinical trials, 11 of them (including those that have been granted an EUA; Table 1) are in phase III trials. These include CoronaVac, inactivated SARS-CoV-2, BBIBP-CorV, BBV152, Ad5 nCoV (approved for use in Chinese military), Sputnik V (Gam-COVID-Vac; approved in Russia), ChAdOx1 nCoV-19, Ad26.COV2.S (JNJ-78436735), mRNA-1273, BNT162b2, and NVX-CoV2373. Although efficacy data from phase III trials with these lead candidate vaccines have not yet been published (except partial data for mRNA-1273 and BNT162b2), efficacy data from phase I/II trials look very promising, with virus NGMAT in the range of 16.6–3906 (Figure 1); only minor adverse effects (e.g., pain at injection site, chills, fever, muscle ache, nausea, and fatigue) were reported in phase I/II trials (reviewed in [41,42]). This portion of this review summarizes efficacy data from phase I/II studies (of CoronaVac, inactivated SARS-CoV-2, BBIBP-CorV, BBV152, Ad5 nCoV, Sputnik V, ChAdOx1 nCoV-19, Ad26.COV2.S, and NVX-CoV2373) and makes recommendations for future studies. Because the correlates of SARS-CoV-2 protection are not well known and it is assumed—based on preclinical studies in nonhuman primates [43] and on successful outcomes following passive transfer of convalescent sera to COVID-19 patients [21,22,23,24]—that SARS-CoV-2-specific neutralizing antibodies are associated with protection, this review will only focus on antibody responses following immunization; the candidate vaccines are reviewed/grouped below based on the approaches that were used to develop them.

### 3.1. Inactivated SARS-CoV-2 Vaccines

#### 3.1.1. CoronaVac

CoronaVac is an inactivated SARS-CoV-2 candidate vaccine (formulated in aluminum hydroxide) developed by Sinovac. In a phase II trial conducted with 600 healthy participants (18–59 years old), more than 92% of the participants immunized with two doses of the vaccine (either 3 or 6 μg/dose, four weeks apart) showed seroconversion, four weeks after a second dose [30]; for the purpose of this review, seroconversion is defined as having a certain value (based on each vaccine trial) of NGMAT above baseline titers. Participants immunized with 3 μg of the vaccine had an NGMAT of 44.1 while those immunized with 6 μg had an NGMAT of 65.4 (Figure 1A). Thus, CoronaVac is immunogenic and elicits a better NGMAT at a higher dose.

In order for the vaccine to meet the features of an ideal SARS-CoV-2 vaccine, the following limitations need to be addressed during phase III studies and/or are recommended during phase IV studies:

(i) Data showing NGMAT in individuals with comorbidities and from different ethnic/racial backgrounds; the phase II trial was conducted only on a healthy group of participants and the ongoing phase III trial (Table 1) excludes individuals with an immunodeficient immune system. Thus, it is not clear how individuals with comorbidities (in dire need of SARS-CoV-2 vaccine) or patients with a compromised immune system will respond to CoronaVac.
(ii) Data showing NGMATs in different age groups. It is not clear whether the immunogenicity of CoronaVac decreases with age as has been observed with other SARS-CoV-2 candidate vaccines [31,33,35,44,45]. In other words, do older adults (50–59 years old) mount a lower response to the vaccine compared to younger adults (18–29 years old)?
(iii) Data demonstrating the immunogenicity of the vaccine in older adults (>60 years, who are in dire need of a SARS-CoV-2 vaccine). We do not know how immunogenic the vaccine will be in people in this age group. If immune responses in the 50–59 years old group are lower than those in the 18–29 years old group, a dose of more than 6 μg/dose of CoronaVac may be required to elicit a strong immune response in adults who are >60 years old. Thus, in phase III trial, the first priority should evaluate 6 μg dose (instead of 3 μg as indicated in Zhang et al., [30]), at 28 days schedule, given the fact that this dose had minor adverse effects (e.g., pain at injection site), which were not different from the 3 μg dose.
(iv) Data showing the longevity of neutralizing antibody titers beyond 28 days; phase II neutralizing antibody titers were conducted using sera collected 28 days (after the second dose). Thus, it is not clear how long the neutralizing antibody titers will last.
(v) Assess the efficacy and safety of the vaccine in an age group, which is 9–17 years old. As mentioned above, CoronaVac is an inactivated vaccine formulated with the adjuvant, aluminum hydroxide; unfortunately, some inactivated viral vaccines against respiratory diseases (e.g., respiratory syncytia virus [46]) formulated with aluminum adjuvant have been associated, in a few cases, with vaccine-associated enhanced viral disease such as pulmonary immunopathology. Although pulmonary immunopathology, in non-human primates or human primates, has not been reported for CoronaVac or any SARS-CoV-2 vaccine, it has been reported in preclinical studies with SARS-CoV and MERS [47].
(vi) Assess the potential for long-term adverse effect(s). Preclinical studies in non-human primates did not show that CoronaVac can promote ADE [48]. However, it is not known whether the same will be true once antibody titers wane.
(vii) Determine whether the NGMATs (44.1 and 65.4) elicited by the vaccine will offer efficient protection. Preclinical studies of the vaccine with non-human primates showed that a lower NGMAT (of 24) offers complete protection from SARS-CoV-2 infection [48]; however, the titers in clinical trials (44.1 and 65.4) are lower than those in convalescent sera from patients who have recovered from COVID-19 (with NGMAT of at least 70) [33,49,50].
(viii) Data showing the efficacy of the vaccine in a larger number of participants. Phase II trials were conducted only with 600 participants, which is a very small proportion of the general population.

#### 3.1.2. Inactivated SARS-CoV-2 and BBIBP-CorV

Inactivated SARS-CoV-2 and BBIBP-CorV are candidate vaccines developed by Sinopharm. In an interim phase II trial conducted with 224 healthy participants (18–59 years old), ~97.6% of participants immunized with two doses of inactivated SARS-CoV-2 strain WIV04 (5 μg/dose, two or three weeks apart) showed seroconversion, two weeks after a second dose. NGMATs were 121 (when immunized two weeks apart) and increased to 247 (when immunized three weeks apart); Figure 1A [51].

For the BBIBP-CorV candidate vaccine (inactivated strain SARS-CoV-2 HB02), in a phase I/II trial conducted with 192 healthy participants (18–80 years old), 100% of participants immunized with two doses of either 2, 4, or 8 μg/dose (four weeks apart) seroconverted, two weeks after the second dose [44]. NGMATs in the 18–59 years old group immunized with either 2, 4, or 8 μg were 87.7, 211.2, and 228.7, respectively, two weeks after the second dose. On the other hand, 60–80-year-old participants immunized with the same doses/schedule had lower NGMATs: 80.7, 131.5, and 170.9, respectively. Neutralizing antibody titers against ten strains of SARS-CoV-2 (56Y, 834Y, HN97, F13, HB01, BJ01, CQ01, QD01, passage 7 stock of strain04,QD01, etc.) using sera from the 4 μg immunized group were also tested and the titers ranged from 117.4 to 394.8 (two weeks after the second dose). Strains 56Y, 834Y, BJ01 are variants with D614G in the spike protein.

In another study, participants (18–59 years old) were immunized at three weeks apart (instead of four weeks as above) with two doses of BBIBP-CorV (4 μg/dose). In this group, NGMAT increased to 282.7, four weeks after the second dose. Thus, two doses of 4 μg of BBIBP-CorV (three weeks apart) elicit higher NGMAT compared to immunization with the same dose at two weeks interval. The former schedule is therefore recommended for BBIBP-CorV vaccine and it is in line with ongoing phase III trials. It is also recommended that the limitations identified above (for CoronaVac) be addressed during phase III and/or phase IV studies of inactivated SARS-CoV-2 strain WIV04 and BBIBP-CorV vaccines. In addition to these, the immunogenicity of BBIBP-CorV at a higher dose (8 μg/dose, three weeks apart) in 60–80-year-old participants should be assessed; based on the data above, with 4 μg of inactivated SARS-CoV-2 strain WIV04 in this age group, it is unlikely that 4 μg of BBIBP-CorV will elicit antibodies with NGMAT > 200 in this age group.

### 3.2. Replication Deficient Vector Vaccines

#### 3.2.1. Ad5 nCoV

Ad5 nCoV is a replication-deficient adenovirus (Ad) type 5 virus particle expressing a full length of the spike protein that was developed by CanSino. In a phase II trial conducted with 508 healthy participants (18–54 years old), 47% and 59% of participants immunized with a single dose of 5 × 10^10^ and 1 × 10^11^ Ad5 nCoV virus particles, respectively, showed seroconversion, four weeks after a second dose [31]. Adults aged 18–44 years old, immunized with 5 × 10^10^ or 1 × 10^11^ virus particles, had NGMATs of 21.2 and 24.6, respectively (Figure 1A). However, NGMATs decreased with age; older adults, 45–54 years old, immunized with 5 × 10^10^ and 1 × 10^11^ virus particles, had NGMATs of 17.8 and 16.6, respectively. Participants who had pre-existing anti-Ad5 antibody titers ≤ 200 and were immunized with 5 × 10^10^ virus particles had an NGMAT of 27 while participants with anti-Ad5 antibody titers > 200 had an NGMAT of 13.8 (four weeks after immunization). A similar trend was observed following immunization with 1 × 10^11^ virus particles; participants who had pre-existing anti-Ad5 antibody titers ≤ 200 and were immunized with 1 × 10^11^ virus particles had an NGMAT of 31.2 while participants with anti-Ad5 antibody titers > 200 had NGMATs of 12.2 (~2.5-fold less). Thus, pre-existing anti-Ad5 antibodies (especially at titers > 200) have a negative effect on the immunogenicity of the vaccine. Unfortunately, a majority of the human population (65–100% of Africans, 30–80% of Asians, 61% of Europeans, and 37–70% of Americans) already have pre-existing anti-Ad5 antibodies (from previous infections with Ad5) with neutralizing antibody titers—in some cases, >1000 [52,53,54]. Consequently, the Ad5 nCoV vaccine may not offer effective protection in this group of people including older adults (45–54 years old and beyond). For Ad5 nCoV to be an ideal SARS-CoV-2 vaccine, it is recommended that the limitations identified above (for CoronaVac) should be addressed during phase III and/or phase IV studies. In addition to those recommendations, a booster dose is recommended, especially in individuals ≥45 years; it is not clear whether the NGMATs (16.6 and 17.8), which are lower than those in convalescent sera from patients who have recovered from COVID-19 [49], will be protective. If a booster dose is needed, the interval(s) to boost needs to be determined given the fact that overall anti-Ad5-neutralizing antibody titers after one immunization were ~700 (an ~3.8-fold increase); high titers of anti-Ad5-neutralizing antibodies may attenuate boosting and thus it may be necessary to wait for the titers to reduce before a boost is administered. Last but not the least, the efficacy and safety of Ad5 nCoV in the <18 years old age group should be assessed.

#### 3.2.2. Sputnik V (Gam-COVID-Vac)

Sputnik V is a two-component candidate vaccine developed by Gamaleya National Center of Epidemiology and Microbiology. It is composed of Ad26-S and Ad5-S virus particles, which are replication-deficient adenovirus type 26 and 5 virus particles, respectively, each expressing a full length of the spike protein. The vaccine is available in two versions—a frozen and a lyophilized version. In a phase I/II trial conducted with 76 healthy participants (18–60 years old), 100% of participants immunized with one dose of 1 × 10^11^ Ad26-S virus particles followed by the same dose of Ad5-S (three weeks apart) showed seroconversion, three weeks after a second dose [32]. NGMATs (three weeks after boost) were 49.25 and 45.95 for frozen and lyophilized versions of the vaccine, respectively (Figure 1A). The lyophilized version of Sputnik V only requires refrigeration (2–8 °C) and thus it has the potential to serve remote regions, which lack freezing facilities for transportation and storage of the frozen version of the vaccine. For Sputnik V to be an ideal SARS-CoV-2 vaccine, it is recommended that the limitations identified above (for CoronaVac) should be addressed during phase III and/or phase IV studies. In addition to those, the following are also recommended:

(i) Determine the efficacy of the vaccine in a group with high (>200) pre-existing anti-Ad26 and Ad5-neutralizing antibody titers. Phase I/II studies were conducted in participants with low pre-existing anti-Ad-neutralizing antibody titers (~25); 43–67% of people in some African countries, ~54% in Thailand, and 5.4–17.8% of people in other regions around the world have pre-existing anti-Ad26 antibodies [53]. It is worth noting that some of these people have neutralizing antibody titers of 200–1000.
(ii) It is also recommended that a larger number of participants be included in future studies as well as a control or placebo group; phase I/II studies did not have a control group and the number of participants were very low.
(iii) Assess the efficacy and safety of the vaccine in other age groups, <18 years and >60 years.

#### 3.2.3. ChAdOx1 nCoV-19

ChAdOx1 nCoV-19 is a replication-deficient chimpanzee Ad virus particle expressing a full length of the spike protein, developed by AstraZeneca. In a phase I/II trial conducted with 1077 healthy participants (18–55 years old), 91–100% of participants immunized with a single dose or with two doses of 5 × 10^10^ ChAdOx1 nCoV-19 virus particles (four weeks apart) showed seroconversion, two weeks after the last dose [55]. NGMATs, after a single dose, were 218 and 51 (Figure 1A); the assays were performed by a plaque reduction neutralization test (PRNT_50_) and microneutralization assay (MNA_80_), respectively. Nine participants in the study were boosted and NGMATs increased to 136 (Figure 1A); titers after boost were tested only by MNA_80_. For ChAdOx1 nCoV-19 to be an ideal SARS-CoV-2 vaccine, it is recommended that the limitations identified above (for CoronaVac) should be addressed during phase III and/or phase IV studies. In addition to those, the following are also recommended:

(i) Assess the efficacy of the vaccine in a population that has high levels (>200) of pre-existing anti-ChAdOx1-neutralizing antibody titers. In a phase I/II trial, only one participant had high levels (>200) of pre-existing anti-ChAdOx1-neutralizing antibody titers. Pre-existing antibodies against chimpanzee adenoviruses in the western world are very low and may not affect the efficacy of the vaccine. However, in countries (especially African countries) with natural habitats for chimpanzee, pre-existing antibodies against chimpanzee adenoviruses are high in the human population [56,57]. Thus, the efficacy of the vaccine in this group of individuals is warranted.
(ii) Assess the efficacy of ChAdOx1 nCoV-19 vaccine in age groups, <18 years and >56 years.

#### 3.2.4. Ad26.COV2.S (JNJ-78436735 or Ad26COVS1)

The Ad26.COV2.S candidate vaccine is a replication-deficient Ad-type 26 virus particle expressing a full length of the spike protein, developed by Johnson & Johnson. Phase I/IIa trial was conducted with 402 healthy participants (18–55 years old) and with 394 healthy older adults (≥65 years old); participants were immunized with a single dose of either 5 × 10^10^ or 1 × 10^11^ Ad26.COV2.S virus particles [45]. A total of 92% of the 18–55-year-old immunized participants with either 5 × 10^10^ or 1 × 10^11^ showed seroconversion, four weeks after immunization. In the ≥65 years old group, only 15 participants were tested for seroconversion (the study is ongoing); in these first 15 participants, 100% (6/6) immunized with 5 × 10^10^ virus particles seroconverted while 83% (5/6) immunized with 1 × 10^11^ virus particles seroconverted. NGMATs for 18–55 years old were 214 and 243 following immunization with either 5 × 10^10^ or 1 × 10^11^ virus particles, respectively (Figure 1A). However, the immunogenicity was slightly lower in elderly (≥65 years old); for example, for the participants (6) immunized with 5 × 10^10^ virus particle, the NGMAT in this group was 196. Similarly, for the participants (6) that were immunized with 1 × 10^11^ virus particle, NGMAT was 127. Neutralizing antibody titers after a second dose (8 weeks apart) are pending. In order for Ad26.COV2.S vaccine to meet the features of an ideal SARS-CoV-2 vaccine, it is recommended that the limitations identified above (for CoronaVac) should be addressed during phase III and/or phase IV studies. In addition to those, the following are also recommended:

(i) Assess the efficacy of the vaccine in a population that has high levels (>200) of pre-existing anti-Ad26-neutralizing antibody titers. Seropositivity/titers for Ad26 vector in participants were not reported and thus it is difficult to assess whether the vaccine will be immunogenic in a population with high titer anti-Ad26-neutralizing antibodies.
(ii) It is recommended that a larger number of participants, especially individuals ≥65 years old, be included in future studies.

### 3.3. Recombinant Protein Vaccine

#### NVX-CoV2373

NVX-CoV2373 is a “nanoparticle” of a trimeric full-length recombinant spike protein formulated in Matrix-M1 adjuvant; the vaccine was developed by Novavax. In a phase I trial conducted with 131 healthy participants (18–59 years old), two groups of participants (~28 in each group) immunized twice with a 5 or 25 μg/dose of NVX-CoV2373 had NGMATs of 3906 and 3305, respectively (Figure 1B) [50]; the second dose was given three weeks after the first dose and neutralization assays were conducted two weeks after the second immunization. The titers were superior to those in convalescent sera that were derived from outpatient symptomatic COVID-19 patients (NGMAT of 837) or asymptomatic COVID-19 patients (NGMAT of 254). However, the NGMAT were lower compared to those in sera of hospitalized COVID-19 patients (NGMAT of 7457). In summary, NVX-CoV2373 is immunogenic. For NVX-CoV2373 to be an ideal SARS-CoV-2 vaccine, it is recommended that the limitations identified above (for CoronaVac) should be addressed during phase III and/or phase IV studies.

## 4. Outlook and Perspectives for the Future

Two SARS-CoV-2 candidate vaccines have been granted an EUA—BNT162b2 in the United Kingdom, Canada, the U.S., the European Union, the United Arab Emirates, Saudi Arabia, etc., while mRNA-1273 has been granted an EUA in the U.S. These candidate vaccines (including others in phase III trials) are not interexchangeable, i.e., if someone receives BNT162b2 as the first dose, they cannot receive mRNA-1273 as the second dose and vice versa. Two other candidate vaccines, Sputnik V and Ad5 nCoV, have been approved in Russia and in China (for the military), respectively, while phase III trials are ongoing. In addition to these, other candidate vaccines are in phase III trials. These candidate vaccines are a testament to the fact that the scientific community and the biotechnology industries have been working relentlessly since this pandemic started. Although a lot of progress has been made in developing and testing these vaccines, there are still unanswered questions; for example, do these lead candidate vaccines meet the features of an ideal SARS-CoV-2 vaccine? As mentioned about, an ideal SARS-CoV-2 vaccine should be highly immunogenic; elicit long-lasting protective antibodies; should be used to immunize everyone regardless of comorbidity or age, immune status, pregnancy/breastfeeding status; should lack the potential to cause ADE or pulmonary immunopathology; and should be thermostable.

Data so far show that these vaccines are very safe; most participants in clinical trials have experienced only minor adverse effects that were short lived and included pain at injection site, chills, fever, muscle ache, nausea, and fatigue (reviewed in [41,42]). In addition to these, a very small number of vaccinees (with a history of severe allergic reactions) who have received BNT162b2, have developed anaphylaxis. ADE, pulmonary immunopathology, or other complications have not been reported in any of the clinal trials or vaccinees; nevertheless, it is recommended that vaccinees should be monitored for at least a year for any signs of vaccine-associated disease. Additionally, the longevity of protection for these vaccines should also be monitored for at least two years. So far, clinical trials have only published data showing neutralization efficacy, two–four weeks after the last dose; thus, it is not known whether the antibodies will wane overtime and, if they do, what would be the implication(s), i.e., what would be the level of protection against COVID-19? Would that cause ADE? These are answered questions that need follow-up studies.

With respect to immunogenicity, the vaccines are highly immunogenic and some of them elicit good NGMATs after a single dose. Although NGMATs from phase III trials of the BNT162b2 and mRNA-1273 vaccines have not yet been published, NGMATs from their phase I/II trials including those of other candidate vaccines (currently in phase III trial; Table 1) look very promising. The viral neutralizing titers differ among candidate vaccines and range from a low of 16.6 to a high of 3906 (Figure 1; NGMAT from clinical trials of BBV152 vaccine have not yet been published). The effect(s) of differences in these NGMATs, between each of these vaccines, in protecting against SARS-CoV-2 infection and/or COVID-19, in the long run, is not known. Thus, it is advisable for each vaccine trial to continue to monitor its participants, by conducting bi-weekly SARS-CoV-2 nucleic acid tests, for at least one year to assess the number of COVID-19 cases (if any) that may be present in a vaccine group versus a placebo group. It is also recommended that the immunogenicity/efficacy of most of the adenovirus vectored candidate vaccines (Ad5 nCoV, Sputnik V, ChAdOx1 nCoV-19, Ad26.COV2.S) be tested in continents, Africa and Asia, which have people with high titers (>200) of pre-existing anti-vector neutralizing antibodies; otherwise, the vaccines may not be applicable to people living in these continents.

While efforts have been made, so far, to assess the immunogenicity of all these vaccines in age groups that are >18 years old, none of the ongoing phase III trials, except with the BTN162b2 vaccine, have included participants who are <18 years old. Thus, the efficacy of most these vaccines in this age group (12–17 years old) and in children is not known. Although children and teenagers are less susceptible to COVID-19 compared to older adults, they can serve as carriers for transmission to older adults. Thus, the efficacy of the vaccines should be assessed in these age groups; vaccinating children and teenagers will be very crucial in fighting the pandemic. Another limitation with most of the vaccine studies is lack of participants with comorbidities. Most of the vaccine studies excluded participants who are obese or have a history of SARS-CoV-2 infection. Thus, it is not known how immunogenic these vaccines will be in this group of people, especially in people with pre-existing antibodies against SARS-CoV-2. At the moment, interim guidelines from the U.S. Centers for Disease Control and Prevention (CDC) indicate that patients with prior SARS-CoV-2 infection (symptomatic or asymptomatic but not current infections) should be vaccinated with BNT162b2 and mRNA-1273 [58]; the CDC indicates that these two candidate vaccines should be given to this group towards the end of 90 days of a documented infection. The CDC also suggests that patients who received convalescent sera or investigational neutralizing monoclonal antibody for COVID-19 treatment should defer vaccination for at least 90 days, until additional data are available. Thus, studies are needed to assess the effect of pre-existing SARS-CoV-2 antibodies to these vaccines.

An ideal SARS-CoV-2 vaccine should be used to immunize everyone including women who are pregnant or breastfeeding as well as people with HIV, have a deficient immune system, or are taking immunosuppressive drugs. While data from immunosuppressed hamsters show that Sputnik V vaccine can offer 100% protection from SARS-CoV-2 [32], the vaccine and others have not been tested in people with a deficient immune system, taking immunosuppressive drugs, with an autoimmune disease, or people who are pregnant/breastfeeding. Thus, the immunogenicity of these vaccines in these groups of people is not known. It is recommended that clinical trials should include people from these groups, especially trials with the two vaccines (mRNA-1273 and BNT162b2) that are based on an mRNA technology, which has never been used in the past to develop a licensed vaccine.

The temperature at which the vaccines will be transported and stored at is also very important. Nine of the 11 candidate vaccines (CoronaVac, Inactivated SARS-CoV-2, BBIBP-CorV, BBV152, Ad5 nCoV, Sputnik V, ChAdOx1 nCoV-19, Ad26.COV2.S, and NVX-CoV2373) require refrigeration at 2–8 °C. Most countries and some remote regions around the world have refrigeration facilities (2–8 °C) for vaccine storage and transportation; thus, there is not going to be a major challenge in transporting and storing these vaccines. However, care must be taken not to accidentally expose the vaccines to freezing conditions; exposure to freezing conditions may affect the efficacy of the vaccines, as has been observed in remote regions with hepatitis B virus vaccine and other vaccines [59,60]. On the other hand, two vaccines (mRNA vaccines; mRNA-1273 and BNT162b2) require freezing conditions; mRNA-1273 requires transportation and storage at temperatures between −25 and −15 °C, while BNT162b2 requires transportation and storage at −70 °C ± 10 °C. Pfizer has indicated that BNT162b2 can be stored at 2–8 °C for up to 5 days and that there are measures in place, GPS-enabled thermal sensors, to track the location and the temperature of the vaccines during shipping. However, these features/capabilities are not readily available in most countries, especially developing countries. Thus, the BNT162b2 vaccine is going to face major challenges with transportation, storage, and distribution in most countries around the world. To enhance worldwide distribution of the vaccine and to reduce the cost (due to refrigeration or freezing) that will be associated with transporting/storing the vaccine, it is recommended that the vaccine be formulated (by desiccation or spray-freeze drying) into a product that is thermostable at room temperature for a longer period of time; a desiccated RNA [61] and a spray-freeze dried candidate vaccine against human papillomaviruses [62,63,64] have been shown to be thermostable at room temperature for up to a year.

## 5. Limitations in Efficacy Data between Vaccine Trials

As mentioned above, the viral neutralizing titers differ among candidate vaccines and range from a low of 16.6 to a high of 3906 (Figure 1). It must be cautioned that the NGMAT data summarized in the figure should not be used to predict the efficacy of a vaccine relative to another, for the following reasons: (i) the doses of the antigens used for immunizations were different between vaccines and participants were immunized at different schedules (two to four weeks apart). (ii) The time frames the sera were collected for neutralization assays were different; some studies collected sera one week after the last immunization, while others collected sera two weeks after the last immunization. (iii) Different assays were used (PRNT versus MNA) in determining NGMATs between vaccines; different assays even with the same vaccine study can give different NGMATs. For example, in ChAdOx1 nCoV-19 vaccine study, PRNT and MNA were used to determine NGMATs in a group of participants and the titers were different between assays; NGMATs using PRNT and MNA were 218 and 51, respectively [55]. (iv) The percent cutoffs (PRNT_50_, PRNT_80_, MNA_80_, inhibitory concentration 80%, inhibitory concentration 99%) that were used for neutralization assays (to consider neutralization) were different among vaccine studies. (v) Some assays used wild-type SARS-CoV-2 for neutralization assays while others used a SARS-CoV-2 reporter virus expressing mNeonGreen. The above differences are, therefore, limitations when comparing NGMATs and efficacies between vaccines.

It must also be pointed out that, in this review, it was mentioned that some candidate vaccines elicited NGMATs that were higher than those in convalescent sera from patients who have recovered from COVID-19 and vice versa. This information also has to be interpreted with caution and may not be used to compare the efficacy of one vaccine to another, for the following reasons: (i) convalescent sera used for different vaccine studies were collected from patients at different time points following exposure and recovery from COVID-19; neutralizing antibodies in COVID-19 patients peaks at 15 days after infection [65] and start to decrease 2–3 months post-infection [66]. Sera collected outside this time window and used in vaccine studies may have low levels of neutralizing antibodies. (ii) The age, sex, and duration of infection (symptomatic, asymptomatic, and hospitalization) in the people from whom the convalescent sera were collected were different. Younger COVID-19 patients, female patients, and outpatient COVID-19 patients seem to have lower antibodies compared to older COVID-19 patients, male patients, and hospitalized patients [33,50,65]. Thus, sera collected from these different groups in vaccine studies are going to have different antibody titers. (iii) The type of neutralization assays that were used to determine NGMATs in convalescent sera differed from one vaccine study to another.

In summary, the SARS-CoV-2 candidate vaccines (with an approved EUA after phase III trials) including those that are still undergoing phase III trials are very safe, immunogenic, and elicit good neutralizing antibody responses. Although each of the candidate vaccines has a limitation, which does not make the vaccine “an ideal vaccine” at the moment, they can be used to fight the pandemic. For the pandemic to be controlled, a mass vaccination campaign, worldwide, has to be initiated. Because SARS-CoV-2 is very contagious and because highly contagious infectious agents normally require >90% of immunity in a population to confer herd immunity, it is likely that a higher proportion of the world’s population has to be vaccinated in order to protect those that will not be vaccinated; fortunately, recent polls show that an increasing number of people (especially in the U.S.), 71% in November/December versus 63% in August/September, are willing to get vaccinated against the virus [67]. Nevertheless, the percentage of the world’s population that needs to be vaccinated in order to prevent the pandemic is unknown. Another unknown with SARS-CoV-2 is whether people who will be vaccinated against the virus (in the coming weeks or months) need to be re-vaccinated after a certain time frame or yearly like influenza viruses. The mutation rate of SARS-CoV-2 (~10^−3^ substitutions/site/year [68]) is only approximately half that of influenza viruses (2.6 × 10^−3^ substitutions/site/year [69]). Nevertheless, it is worth investigating whether the vaccines in development can protect against current and future variants of SARS-CoV-2. This is very important given the fact that, in less than one year of emergence, so many variants of the virus, especially in the spike protein (A222V, D614G, D839Y/N, D936Y/H, P1263L, etc.), have been reported worldwide [70,71]; some of these variants, especially D614G, have been shown to be associated with increased infectivity [71] while others (G476S and V367F) are resistant to neutralization by monoclonal antibodies [72]. In addition to these, a new variant with mutations (deletions at amino acids 69, 70 and 144; substitutions N501Y, A570D, D614G, P681H, T716I, S982A, and D1118H), in the S, that make SARS-CoV-2 highly contagious has emerged in the United Kingdom [73]; it is not clear whether the candidate vaccines with approved EUA or in clinical trials can protect against this variant. Thus, more neutralization studies are needed to study this new variant and others as they emerge. So far, a few candidate vaccines (mRNA-1273 and BBIBP-CorV) have assessed efficacy against some SARS-CoV-2 variants and the neutralizing antibody titers look promising [36,44].

The mRNA technology that was used to develop BNT162b2 and mRNA-1273 candidate vaccines look very promising; the technology has so many advantages over conventional vaccine approaches (e.g., inactivated vaccines and recombinant protein-based vaccines). For example, once immunized with the vaccine, the mRNA can be translated for a long period of time, “on site” in the body, thus saving time/costs that would have been associated with expressing and purifying the proteins (in the correct conformation) in the lab. In addition to this, the spike protein, once expressed in the body, is post-translationally modified, thus circumventing post-translational modification-related issues associated with proteins expressed in the lab (some of which can affect immunogenicity). The technology is also advantageous over replication deficient adenovirus vector vaccines that carry the DNA of the spike protein. With mRNA vaccines, the RNA is translated directly in the cytoplasm; however, with replication deficient vector vaccines, the spike protein DNA must be delivered into the nucleus for transcription prior to translation. Furthermore, mRNA vaccines do not have pre-existing anti-mRNA antibodies in the body unlike replication deficient adenovirus vector vaccines, which have pre-existing anti-adenovirus antibodies in the human population; these antibodies will attenuate the immunogenicity of the vaccines. That being said, mRNA vaccines have some challenges. For example, BNT162b2 and mRNA-1273 are less stable and thus they must be transported and stored frozen. Further, mRNA can interact with toll-like receptors in the cells leading to the production of pro-inflammatory cytokines and type I interferons with dangerous consequences. In addition to these, the mRNAs are encapsidated in lipid nanoparticles (LNPs). LNPs can be toxic (if they accumulate in the liver) and they can activate B cells in the spleen to elicit antibodies against polyethylene glycol (PEG, is a component of the vaccines); anti-PEG antibodies are associated with anaphylactic or hypersensitive reactions (complement activated related pseudoallergy) [74,75]. Most recently, a few people (with a history of severe allergic reactions) vaccinated with the BNT162b2 candidate vaccine developed allergic reactions within 24 h of vaccination. It is not clear what might have caused the allergic reactions; the LNPs in the vaccine are composed of lipids ((4-hydroxybutyl)azanediyl)bis(hexane-6,1-diyl)bis(2-hexyldecanoate), 2 [PEG-2000]-N,N-ditetradecylacetamide, 1,2-distearoyl-sn-glycero-3-phosphocholine, and cholesterol). In addition to this, the vaccine contains the following salts: potassium chloride, monobasic potassium phosphate, sodium chloride, dibasic sodium phosphate dihydrate, and sucrose. Any of these components could have caused the allergic reactions. Thus, studies are needed to study the safety/immunogenicity of these RNA vaccines in individuals with severe allergies. Additionally, the long-term effect of these RNA candidate vaccines in vaccinees needs to be evaluated given the fact that this vaccine technology has never been used to develop a licensed vaccine.

## Figures and Tables

**Table 1:** Lead SARS-CoV-2 candidate vaccines with EUA granted, in phase III trials, or licensed (prior to phase III trials).

Developer	Vaccine Name (Component)	Storage Temp. ^ϕ^	Required Doses	Weeks Apart	Age Group (Years)	Exclusion Criteria from Studies	Clinical Trial Registration Numbers
**Inactivated Vaccines**							
Sinovac	CoronaVac (inactivated SARS-CoV-2 in aluminum hydroxide adjuvant)	2–8 °C	2x: 600 SU/dose *	2 and 4	18–59	-History of COVID-19 or presence of SARS-CoV-2 antibodies -Pregnant/breastfeeding -Used immunosuppressive agents within 6 months -Immunodeficient (HIV)	NCT04582344 NCT04508075
Sinopharm	Inactivated SARS-CoV-2 (inactivated SARS-CoV-2, strain WIV04, in aluminum hydroxide adjuvant)	2–8 °C	2x (dosage not provided)	3	≥18–85	-History of SARS-CoV-2, SARS-CoV or MERS-CoV infections -Pregnant -Received other investigational CoV vaccines (SARS-CoV and MERS-CoV) -Immunodeficient (HIV) -Receiving anti-TB therapy	NCT04510207 NCT04560881 NCT04510207 NCT04612972
	BBIBP-CorV (inactivated SARS-CoV-2, strain HB02, in aluminum hydroxide adjuvant)						
Bharat Biotech	BBV152 (inactivated SARS-CoV-2 in aluminum hydroxide gel—imidazoquinoline adjuvant)	2–8 °C	2x: 6 μg/dose	4	≥18	-History of SARS-CoV infection -History of COVID-19 investigational or licensed vaccination -Pregnant/breastfeeding -Immunodeficient (HIV) -Hepatitis B or C infection	NCT04641481
**Replication Deficient Vector Vaccines**							
CanSino	Ad5 nCoV ^%^ (replication-deficient Ad type 5 vector expressing full-length spike protein)	2–8 °C	1x (VP not provided) **	N/A	≥18	-History of COVID-19 or presence of SARS-CoV-2 antibodies -Pregnant/breastfeeding -Using immunosuppressive agents or immunodeficient -Adenovirus vectored vaccines	NCT04526990 NCT04540419
Gamaleya National Center of Epidemiology and Microbiology	Sputnik V ^€,#^ or Gam-COVID-Vac (combined replication-deficient Ad types 5 and 26 vectors each expressing full-length spike protein)	Frozen version (−18 °C) and lyophilized version (2–8 °C)	2x (prime with rAd26-S and boost with rAd5-S): VP dosage not provided ***	3	≥18	-History of COVID-19 or presence of SARS-CoV-2 antibodies -Pregnant/breastfeeding -Immunosuppressive agents within 3 months -Immunodeficient (HIV) -Tuberculosis	NCT04564716 NCT04530396
AstraZeneca	ChAdOx1 nCoV-19 (replication-deficient Ad type 5 vector expressing full-length spike protein)	2–8 °C	1x: 5 × 10^10^ VP 2x: 5 × 10^10^ VP and 3.5–6.5 × 10^10^ VP	4-12	18–55 56–69 ≥70	-History of COVID-19 or presence of SARS-CoV-2 antibodies -Pregnant/breastfeeding	NCT04536051 NCT04516746 NCT04540393 NCT04400838
Johnson & Johnson/Janssen Pharma	Ad26.COV2.S or JNJ-78436735 (replication-deficient Ad-type 26 vector expressing full-length spike protein)	2–8 °C	1x: 5 × 10^10^ VP	N/A	≥18	-Previous vaccination with CoV vaccine -Received investigational adenoviral-vectored vaccines within 6 months	NCT04505722
**mRNA Vaccines**							
Moderna	mRNA-1273 ^∑^ (mRNA of full-length spike protein in a lipid nanoparticle)	Frozen between −25 and −15 °C	2x: 100 μg each	4	≥18	-History of SARS-CoV-2 infection -Pregnant/breastfeeding -Received other investigational CoV vaccines (SARS-CoV and MERS-CoV) -Received systemic Immunosuppressants for >14 days within 6 months	NCT04470427
Pfizer and BioNTech	BNT162b2 ^∑^ (mRNA of full-length spike protein in a lipid nanoparticle)	Frozen at −70 °C ± 10 °C	2x: 30 μg each	3	≥12	-Symptoms/diagnosis of COVID 19 by NAAT ^$^ -Pregnant/breastfeeding -Immunocompromised -Previous vaccination with CoV vaccine -Immunosuppressive therapy Note: Positive serological test for SARS-CoV-2 was not excluded	NCT04368728 Protocol C4591001
**Recombinant Protein Vaccine**							
Novavax	NVX-CoV2373 (a “nanoparticle” of trimeric full-length recombinant spike protein formulated in Matrix-M1 adjuvant)	2–8 °C	2x: 5 µg SARS-CoV-2 rS + 50 µg Matrix-M1 adjuvant/dose	3	18–84	-History of COVID-19 or presence of SARS-CoV-2 antibodies -Pregnant/breastfeeding -Immunosuppressive or immunodeficient status	NCT04583995 NCT04611802

^ϕ^ Storage or transportation temperature. * SU not defined in trial. A amount of 3 µg was proposed for a phase III trial [30]. 1x = 1 immunization. 2x = 2 immunizations. VP = virus particles. NA = not applicable. ** Phase I/II studies used two different doses: 5 × 10^10^ and 1 × 10^11^ virus particles [31]. *** Phase I/II studies used 1 × 10^11^ virus particles/dose [32]. ^#^ Two versions are available: a frozen version and a lyophilized version, Gam-COVID-Vac-lyo, to be reconstituted. ^%^ Approved for use in Chinese military. ^€^ Approved for use in Russia. ^∑^ EUA granted. ^$^ *NAAT* = nucleic acid amplification test.
